# Young adults’ work-family life courses and mental health trajectories from adolescence to young adulthood: a TRAILS study

**DOI:** 10.1007/s00127-024-02664-8

**Published:** 2024-03-29

**Authors:** Vendula Machů, Iris Arends, Josué Almansa, Karin Veldman, Ute Bültmann

**Affiliations:** 1 Department of Health Sciences, Community and Occupational Medicine, University Medical Center Groningen, University of Groningen, Groningen, The Netherlands; 2 Arbo Unie, Utrecht, the Netherlands

**Keywords:** Internalising problems, Externalising problems, Work, Family, Cohort study, Young adulthood

## Abstract

**Purpose:**

Work-family life courses have been associated with mental health at various time points in life but little is known about how mental health develops during these work-family life courses. The aim of this study was to examine mental health trajectories from adolescence to young adulthood in women and men with different work-family life courses.

**Methods:**

Data from 992 young adults participating in the 18-year follow-up TRacking Adolescents’ Individual Lives Survey (TRAILS) were used. Work-family life courses from ages 18 to 28 years were previously constructed using sequence analysis. For each work-family life course, trajectories of internalising and externalising problems from ages 11 to 29 years were estimated using a multi-group random intercept growth model. Differences in mental health trajectories were examined across work-family life courses.

**Results:**

For women, trajectories of internalising and externalising problems in young adulthood differed significantly between work-family life courses (*p* = 0.037 and *p* < 0.001, respectively). Women in the *inactive* work-family life course reported the highest scores of internalising and externalising problems during the entire young adulthood but the differences in mental health scores became most pronounced at age 29. Trajectories of internalising and externalising problems of men did not significantly differ between the work-family life courses.

**Conclusion:**

Mental health trajectories differed between women depending on their work-family life course. In men, differences between work-family life courses were less pronounced. Future studies should examine which work-family events and transitions captured in work-family life courses are associated with subsequent mental health problems during longer follow-up.

**Supplementary Information:**

The online version contains supplementary material available at 10.1007/s00127-024-02664-8.

## Introduction

Young adulthood is a demographically dense life stage with several important transitions happening simultaneously in both the work and family domains [1]. Traditionally, five markers of the transition to adulthood have been suggested: finishing education, getting a job, leaving home, getting married and having a child [2]. Although the perception of which events mark the transition to adulthood differs among cultures and develops over time [3], this transitional period may affect young adults’ mental health in various ways. For example, it has been shown that achieving age-specific transitions in both the work and family domains and having the feeling of advancing at this life stage is associated with better well-being and mental health in young adulthood [4]. Also, the simultaneous navigation of new roles in the work and family domains, such as combining full-time employment with a socially active life, being a spouse or a parent, and the frequent changes in these roles can pose a challenge to mental health [5–7]. These challenges underscore the importance of better understanding the development of work and family lives during the transition to adulthood and its association with mental health.

By examining work-family life courses, valuable knowledge can be gained about how young people transition into adulthood and how they combine work and family roles [4, 8–10]. A work-family life course entails the sequencing of work and family events during a person’s life and captures the timing, ordering and duration of such events. Constructing and examining work-family life courses is useful as it allows linking life courses to mental health outcomes, providing insights into which specific life courses are more strongly associated with the experience of mental health problems. Our recent systematic review on work-family life courses showed that, overall, work-family life courses characterised by an early transition into parenthood, single parenthood and weak ties to employment were associated with worse mental health outcomes later in life [11].

Understanding the relationship between work, family and mental health requires not only examining the relationship between work-family life courses and later mental health but also considering mental health both before and during the work-family life courses. Previous studies demonstrated that adolescent mental health problems often have their onset in adolescence and persist into early young adulthood [12]. In our earlier study, we showed that adolescent mental health problems were associated with work-family life courses in young adulthood [13]. Since the differences in mental health between young adults with different work-family life courses exist already before the start of working life, they need to be taken into consideration. To date, only two studies examined how mental health changed over time among young people with different work-family life courses [4, 14]. Amato and Kane [14] showed that depressive symptoms declined between ages 18 to 23, regardless of the work-family life course, in a population of US women born in 1976–1979. Salmelo-Aro et al. [4] found that people in life courses characterised by postponed work and family transitions reported an increase in depressive symptoms from their 20s to their 30s compared with people in other work-family life courses. While these studies showed that mental health changed during the work-family life course, it remains unclear when changes occurred. A more in-depth understanding of how mental health develops before and during the work-family life courses, i.e. in adolescence and in young adulthood, can help to identify the relevant time period when changes occur. This information is useful for determining when interventions aimed at supporting young adults’ mental health may be necessary and most beneficial.

In the present study, we examine whether and how mental health trajectories from adolescence to young adulthood differed among young adults with different work-family life courses. We add to the literature in two ways. First, we include mental health measurements far before the start of work-family life courses (i.e., as of early adolescence). Second, we rely on multiple mental health measurements during the work-family life course to better examine how mental health develops during young adulthood.

## Methods

### Study design and sample

Data were used from the TRacking Adolescents’ Individual Lives Survey (TRAILS), a prospective cohort study with an 18-year follow-up [15, 16]. In 2001, adolescents from five municipalities in the northern part of the Netherlands born between October 1, 1989, and September 30, 1991, were invited to participate in TRAILS. In total, 2230 children (average age 11 years) participated in the first measurement wave. Data were subsequently collected at six follow-up measurement waves at average ages 13.5, 16, 19, 22, 26 and 29 years. Out of the original sample, 1231 participants completed the assessment at the 7th measurement wave. The analytical sample of this study consists of 992 respondents (44.5% of the baseline sample) who reported their work history at the 5th and 7th measurement waves, their educational history at the 7th measurement wave and the age of their oldest child at the 7th measurement wave. Those participants for whom the data was not available (*n* = 239) were more likely to be men, parents, lower educated, and more likely to experience internalising and externalising problems at the 6th and the 7th wave.

### Measures

#### Work-family life courses

Monthly information on education, work and parenthood was derived for 120 months between the 18th and 28th birthdays. We defined four possible states in the work domain combining education and work: in education, in work, in education and in work, not in education nor work. Additionally, we defined two possible states in the family domain: parent and not a parent. Combining the work and family states gave 8 possible work-family states (4 work states x 2 family states). Previously, we derived a typology of six work-family life courses in young adulthood for both women and men by using sequence analysis. Further details on the analytical approach can be found in Machů et al. [13].

Five of the six work-family life courses were similar in women and men and were assigned the titles *long education, continuous education and work, education and work to work, early work* and *inactive.* The sixth work-family life course differed between women and men in the frequency and timing of the transition into parenthood and was therefore titled *active with children* in women and *active* in men. Work-family life courses are described in Table 1, and state distribution plots are presented in the Supplementary Figures S1 and S2. For more details see Machů et al. [13].

**Table 1:** Description and distribution of work-family life courses in women (*n* = 627) and men (*n* = 365)

	Work-family life course	Description	Women, n (%)	Men, n (%)
1	Long education	Longest time spent in education only (on average 40 months in women, 72 months in men) with a later transition into work.	178 (28.4)	48 (13.2)
2	Continuous education and work	Longest time spent combining education and work, on average more than 88 months, with a later transition into work.	62 (9.9)	42 (11.5)
3	Education and work to work	Combining education and work for around 50 months and subsequently transitioning into work only.	113 (18.0)	68 (18.6)
4	Early work	A short period of combining education and work and a relatively early transition into work only, around 90 months on average spent in work without being in education or being a parent.	96 (15.3)	53 (14.5)
5	Inactive	More than half of the observed period spent in inactive states, i.e. not working and not in education. A total of 35.4% of women and 13.9% of men had become parents by age 28.	85 (13.6)	43 (11.8)
6^a^	Active with children	Longest time spent in education or combining education and work, a relatively early transition into parenthood with almost all women having become mothers by age 28.	93 (14.8)	
6^b^	Active	Mostly in work or combining education with work with 25.2% of men having become fathers by age 28.		111 (30.4)

^a^Trajectory only identified in women, ^b^Trajectory only identified in men

#### Mental health

Mental health was assessed by the Youth Self-Report (YSR) [17] at ages 11, 13.5 and 16 and by the Adult Self-Report (ASR) [18] at ages 19, 22, 26 and 29. Out of the 126 items of the original ASR, we used the 112 items that were comparable with the 112 items of the YSR. Two scales were derived from the YSR and ASR: internalising and externalising problems. Internalising problems capture anxious and depressed behaviour, somatic complaints, and withdrawn and depressed behaviour, while externalising problems cover aggressive and delinquent behaviour.

### Statistical analyses

#### Multi-group growth curve modelling

For each work-family life course, we estimated trajectories of internalising and externalising problems by applying a random intercept growth model with a piece-wise categorical time (per wave). By doing so, we split the total variability into the individual person-level variance and the time-specific variance of each measurement. The growth models were estimated via full information maximum likelihood with robust standard errors, considering the missing values within waves as missing at random. The overall low number of missing values (between 0 and 10% at the individual waves; in total 3% of values missing) provides reassurance that its impact on our results is likely minimal. We used a Wald test to compare the trajectories of internalising and externalising problems across work-family life courses. We tested if all the estimated means of the trajectories were equal across groups. Additionally, after inspecting the trajectories of internalising and externalising problems, we conducted post-hoc analyses to test differences in internalising and externalising scores at ages 19 and 29 years (4th and 7th measurement wave, respectively) between work-family life courses with the lowest and the highest average scores. The measurement waves at ages 19 and 29 were selected because they mark the start and end points of the work-family life courses that were used as a grouping variable for mental health trajectories. The effect size (Cohen’s d) of statistically significant differences was computed as the difference between the means of the two groups at ages 19 and 29 divided by the standard deviation of the sample at the respective ages. Parameter estimates of the trajectory models (means, random-intercept variances and residual variances) are reported in the Supplementary Tables S1 and S2 for women, and S3 and S4 for men.

Multi-group growth curve analyses were conducted in MPlus v8.7 [19].

## Results

### Work-family life courses and mental health trajectories in women

**Fig. 1:**
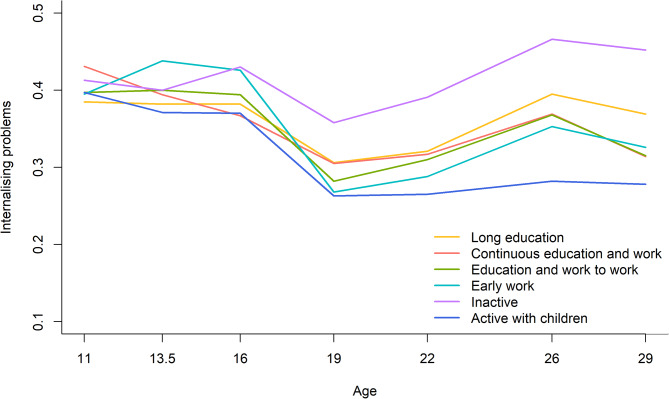
Trajectories of internalising problems across work-family life courses, 11–29 years, women

The trajectories of internalising problems for women in the six work-family life courses are presented in Fig. 1. Women in all work-family life courses showed a decrease in scores of internalising problems during adolescence followed by a slight increase during young adulthood. Women in the *inactive* work-family life course reported the highest scores of internalising problems from age 19 to 29 years, especially compared with women in the work-family life course *active with children*. Trajectories of internalising problems in young adulthood statistically differed between work-family life courses (*p* = 0.037). Post-hoc analyses showed that differences between the work-family life courses in scores of internalising problems at ages 19 and 29 were statistically significant with women in the work-family life course *inactive* reporting significantly higher scores in comparison with women in the work-family life course *active with children* (0.358 vs. 0.263; Cohen’s d = 0.37; *p* = 0.020 at age 19, and 0.452 vs. 0.278; Cohen’s d = 0.61; *p* < 0.001 at age 29).

**Fig. 2:**
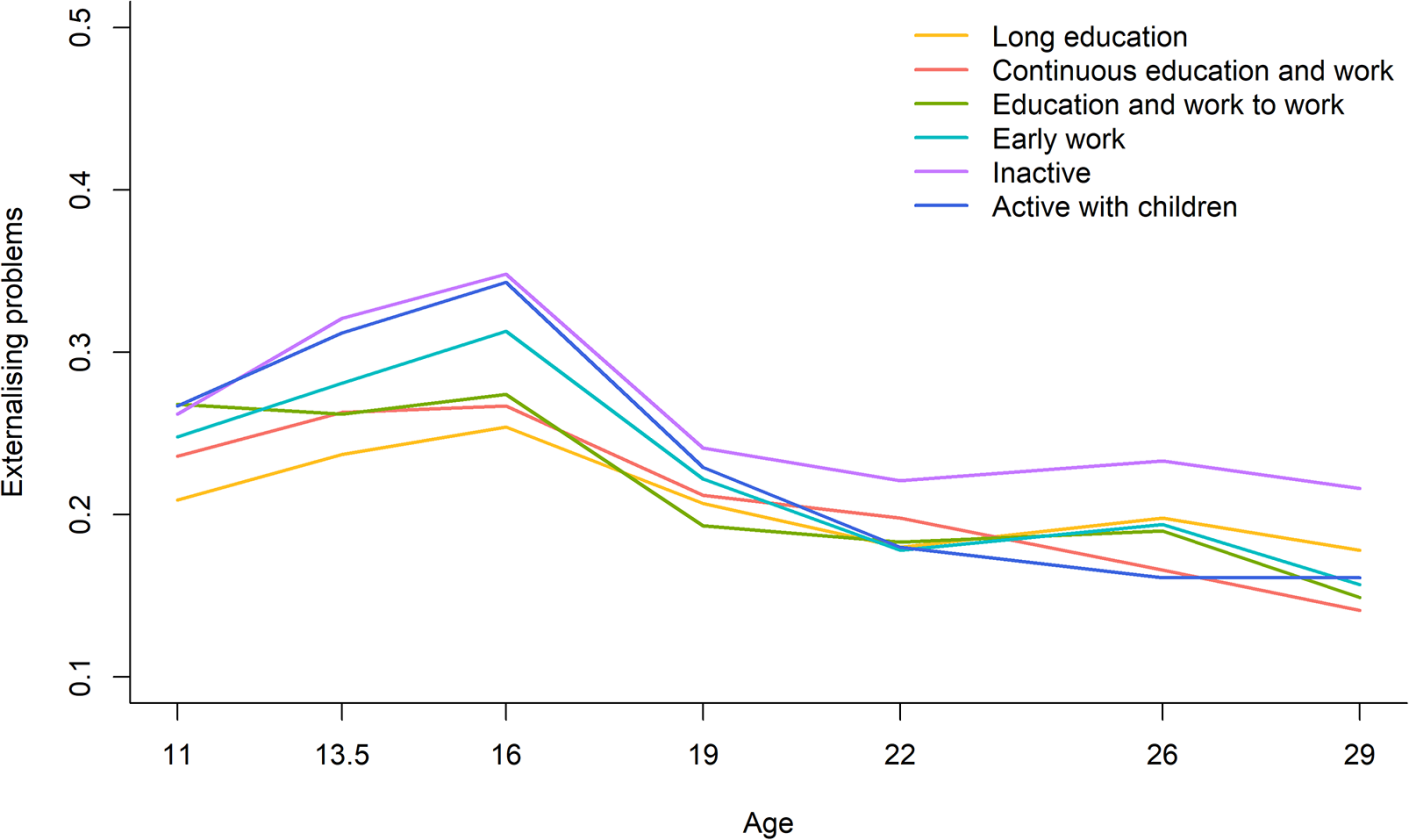
Trajectories of externalising problems across work-family life courses, 11–29 years, women

The trajectories of externalising problems for women in the six work-family life courses are presented in Fig. 2. Externalising problems peaked at age 16 and kept decreasing across all six work-family life courses. Women in the *inactive* work-family life course reported the highest scores of externalising problems from age 19 to 29 years, especially compared with women in the work-family life course *continuous education and work*. Trajectories of externalising problems in young adulthood statistically differed between work-family life courses (*p* < 0.001). Post-hoc analyses showed that differences between the work-family life courses in scores of externalising problems were not statistically significant at age 19 but became statistically significant at age 29. Specifically, at age 29, participants in the work-family life course *inactive* reported significantly higher scores of externalising problems in comparison with those in the work-family life course *continuous education and work* (0.216 vs. 0.141; Cohen’s d = 0.44; *p* = 0.010).

### Work-family life courses and mental health trajectories in men

**Fig. 3:**
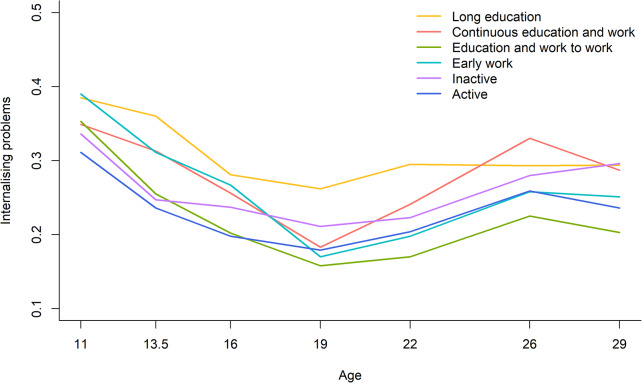
Trajectories of internalising problems across work-family life courses, 11–29 years, men

The trajectories of internalising problems for men in the six work-family life courses are presented in Fig. 3. The trajectories of internalising problems decreased in all work-family life courses from age 11 to 19 and increased from age 19 to 29. Trajectories of internalising problems did not differ significantly between work-family life courses (*p* = 0.662). The post-hoc analyses showed that the significant differences in internalising problems between the work-family life courses *long education* and *education and work to work* at age 19 (0.262 vs. 0.158; Cohen’s d = 0.51; *p* = 0.010) diminished and became non-significant by the age of 29 years.

**Fig. 4:**
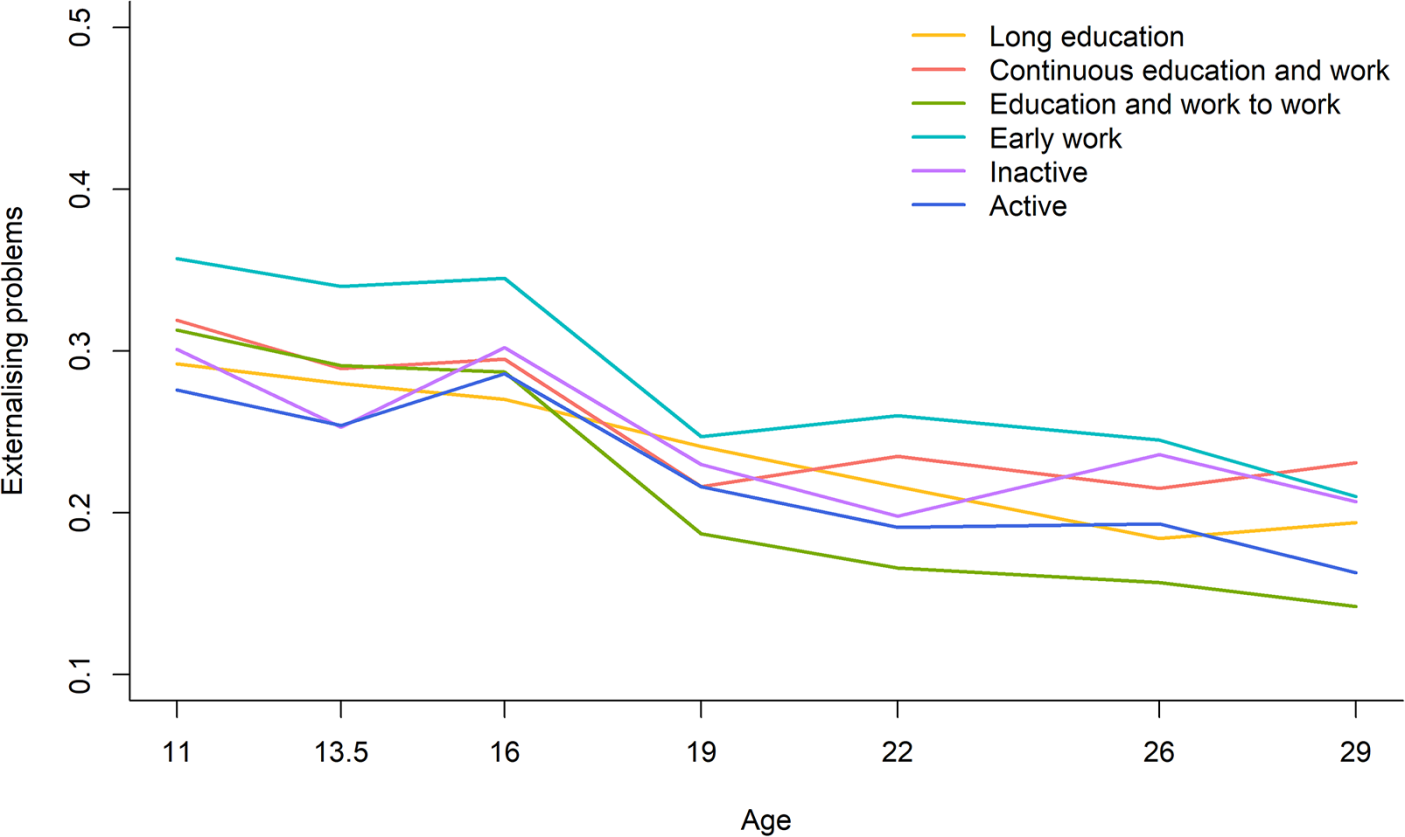
Trajectories of externalising problems across work-family life courses, 11–29 years, men

The trajectories of externalising problems for men in the six work-family life courses are presented in Fig. 4. The trajectories of externalising problems showed a consistent decline from age 11 to 29 in all work-family life courses and they did not differ significantly between work-family life courses (*p* = 0.232). The post-hoc analyses showed that significant differences in externalising problems emerged at the age of 29 years between the work-family life courses *continuous education and work* and *education and work to work* (0.231 vs. 0.142; Cohen’s d = 0.43; *p* = 0.018).

## Discussion

This study examined trajectories of internalising and externalising problems from adolescence to young adulthood in young adults with different work-family life courses. The primary finding is that trajectories of internalising and externalising problems of women in the *inactive* work-family life course diverged from other work-family life courses during young adulthood. Differences in the internalising and externalising problem scores increased during young adulthood with women in the *inactive* work-family life course reporting higher scores in comparison with the work-family life courses *active with children* and *continuous education and work* for internalising and externalising problems respectively.

The *inactive* work-family life course, although not exclusive in experiencing periods of inactivity, represents women who were inactive for over half of the observed period, meaning they were neither in education nor working for more than five years during young adulthood. Our findings on higher scores of mental health problems among the *inactive* work-family life course are in line with previous research showing that being in NEET (not in education, employment or training) in young adulthood is associated with experiencing mental health problems [20]. A potential explanatory mechanism could be that young women with a history of long spells of inactivity do not achieve age-specific transitions such as finishing education or finding a job. Previously, it has been shown that work-family life courses characterised by postponing important transitions during young adulthood, such as a transition from education to work, were associated with worse well-being and mental health in young adulthood [4]. Another explanatory mechanism might be related to the transition to parenthood. One-third of the women in the *inactive* work-family life course became mothers and the combination of inactivity and parenthood may put these women in an especially vulnerable position and decrease their mental health. For instance, a recent study examining the impact of employment on well-being in German mothers showed that their mental health improved after the transition from parental leave into employment [21]. However, the observed relationship between inactivity in young adulthood and higher scores of mental health problems can also operate in reverse, i.e. experiencing mental health problems in adolescence and young adulthood has been shown to decrease participation in educational and employment status [22]. Further research is needed to shed light on mechanisms and causal pathways.

While the *inactive* work-family life course in women displayed higher scores of both internalising and externalising problems compared with the other work-family life courses and the effect sizes of the mean differences were between small and medium [23], the average scores remained below the borderline clinical threshold, i.e. their scores did not meet the criteria for significant mental disorders. Even though the *inactive* work-family life course remained below the borderline threshold, this group of women had consistently higher scores of mental health problems throughout young adulthood, and the difference was at its largest at age 29. For some women in this group, preventive support measures could be beneficial for their future mental health.

The mental health trajectories of all five remaining work-family life courses among women were relatively similar to one another. The overall negligible differences in mental health trajectories between these other five work-family life courses could be a sign of loosening strict expectations of what young adults need to achieve in their 20s. In light of this finding and previous research showing more individualised pathways into adulthood [2], it may not be harmful for mental health to postpone or forfeit some age-specific transitions, as long as spells of inactivity do not dominate the work-family life course. It is also possible that differences in mental health between work-family life courses become larger only later in the life course. A longer follow-up time is needed to examine how the work-family life courses further develop and whether differences in the mental health trajectories emerge later in the life course.

We did not find differences in trajectories of mental health problems between work-family life courses of men. A potential explanation may be that men in a work-family life course labelled as *inactive* may have not exhibited a distinct level of inactivity compared to other life courses. While men in the *inactive* life course spent over half of the observed period in inactive states, their engagement in education or work at the end of young adulthood might have mitigated the potential impact on mental health, making them less distinguishable from the other work-family life courses. Also, the exclusion of men with lower education and prior mental health problems due to the loss to follow-up might limit the representation of inactive men in our sample, potentially underestimating the association between the *inactive* work-family life course and worse mental health. Another explanation may be that men more often underreport mental health problems. Research has shown the problematic impact of conformity to traditional gender norms on underreporting and expression of symptoms of mental health problems among men [24]. Although we did not find differences in mental health trajectories of men, we did observe differences in externalising problems at age 29 between work-family life courses *continuous education and work* and *education and work to work*. The effect size of the mean difference was small (Cohen’s d = 0.43) [23]. Further research is needed to investigate how mental health develops in young men experiencing different education-to-work transitions. Additionally, our study highlights the need to consider gender differences when examining the relationship between work, family, and mental health trajectories during young adulthood.

Finally, the findings of the present study are consistent with and contribute to our previous research in the following ways. Women in work-family life courses characterised by an early transition into parenthood reported on average higher scores of externalising problems during adolescence, and men in the *long education* work-family life course reported higher scores of internalising problems during adolescence, which is in line with the findings from our previous study [13]. A finding emerging from the present study is the higher average score of externalising problems during adolescence among men in the *early work* life course. This is in line with one of our previous studies showing that experiencing externalising problems in adolescence is associated with low or medium educational attainment at age 19 [25]. An important insight provided by this study is the observation of diverging mental health trajectories during young adulthood, particularly in women. Thus, this study underscores the importance of examining mental health problems beyond mere point estimates and considering the developmental trajectory of mental health problems during the life course.

### Strengths and limitations

This study has several strengths. We used detailed information on respondents’ education, work and parenthood histories which made it possible to examine combined work-family life courses during young adulthood. Additionally, we linked mental health trajectories based on data collected at seven mental health assessments spanning a period of 18-year follow-up to the identified work-family life courses. By doing so, we were able to go beyond analysing the associations between work-family life courses and mental health assessed at one or two points in time. This approach allowed us to describe how mental health developed during work-family life courses.

Our findings also need to be interpreted in the light of some limitations. Firstly, our sample comprises 44% of the baseline sample included in TRAILS, resulting in a few of the identified work-family life courses containing fewer than 50 people. Respondents lost to follow-up were most likely to be men, lower educated and had a lower parental SES. Respondents who did not report their work and family histories were more likely to experience internalising and externalising problems at the last two measurement waves. Thus, we might have underestimated the severity of mental health problems in work-family life courses characterised by longer spells of inactivity and lower education due to the loss to follow-up of respondents with more severe mental health problems, especially among men. Secondly, the family domain in our typology of work-family life courses only covers parenthood and does not include other family characteristics such as partnership or residency because detailed data on these aspects was not available. By not including these potentially important predictors of mental health, we may have grouped respondents with substantially different work-family experiences together in the same work-family life course and thus be missing important associations between work-family experiences and mental health.

### Implications

Our findings show that, among women, differences in mental health trajectories between the *inactive* work-family life course and the other life courses started to increase in young adulthood. This indicates an important time window for implementing policies and interventions to support young adults in the *inactive* work-family life course. In order to understand how young people in the *inactive* work-family life course perceive their situation, what their needs are and how they can be supported, qualitative studies are needed. Furthermore, future research may examine whether the timing of key transitions in work-family life courses is associated with changes in mental health. Such research could contribute to identifying entry points for work-family interventions to support young adults’ mental health.

## Conclusions

We showed that there are differences in mental health trajectories from age 11 to 29 between young adult women with different work-family life courses. Women in the *inactive* work-family life course reported the highest scores of internalising and externalising problems during the entire young adulthood. Differences between mental health trajectories started to increase as of age 19 and were largest at age 29. This finding implies that a longer follow-up is needed to examine how trajectories of mental health problems continue to develop beyond young adulthood.

## Electronic supplementary material

Below is the link to the electronic supplementary material.

Supplementary Material 1
