# Supplementary material for: Young adults’ work-family life courses and mental health trajectories from adolescence to young adulthood: a TRAILS study
Source: Soc Psychiatry Psychiatr Epidemiol. 2024 Mar 29;59(12):2227–35. doi: 10.1007/s00127-024-02664-8 (PMC11522154; doi:10.1007/s00127-024-02664-8)
Supplement: Supplementary file 1 — Supplementary Material 1 [file 127_2024_2664_MOESM1_ESM.docx]

Supplementary material

**Supplementary Fig. S1** State distribution plots of work-family trajectories, 18–28 years, women (n = 627)


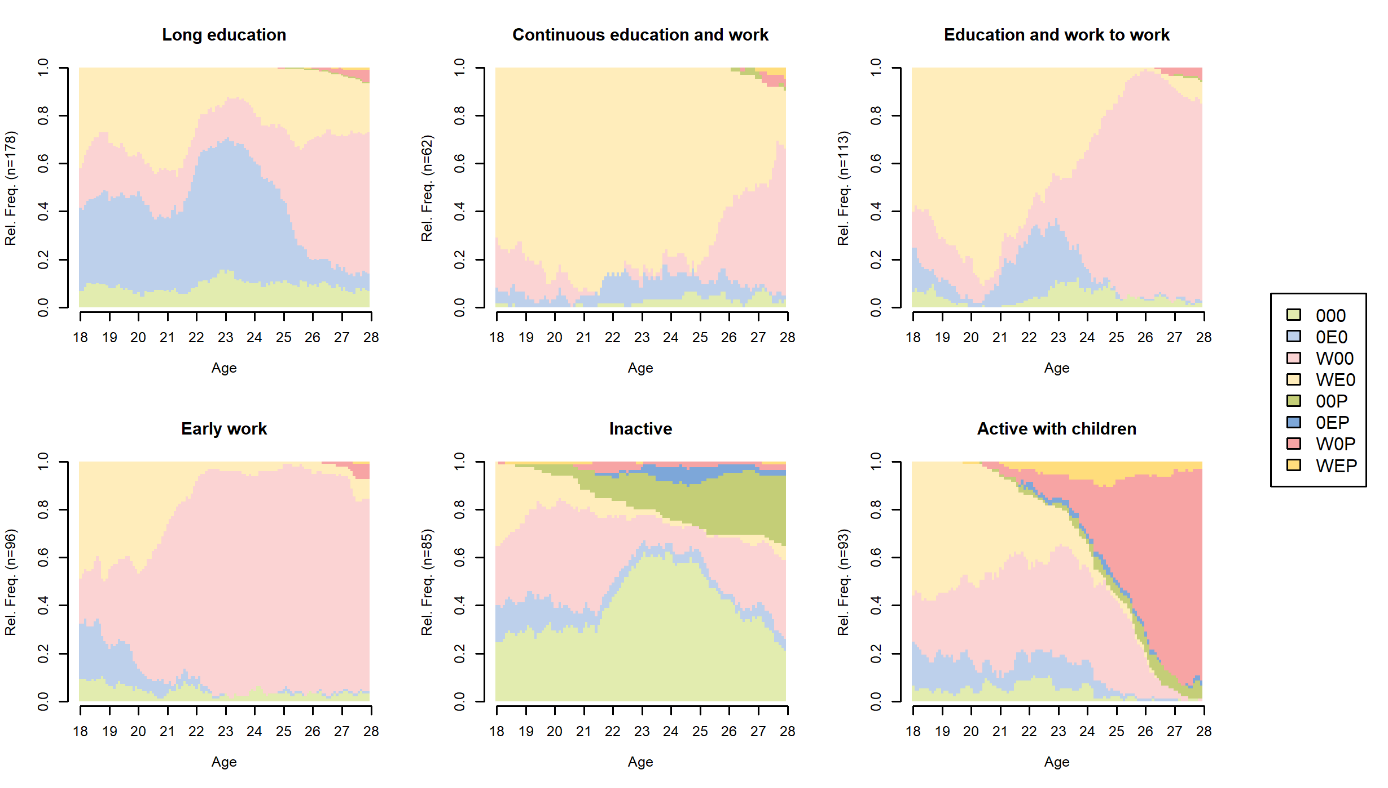


“Fig. 1. State distribution plot of work-family trajectories, 18–28 years, women (n = 627)”. From Machů, V., Veldman, K., Arends, I., & Bültmann, U. (2022). Work-family trajectories in young adulthood: Associations with mental health problems in adolescence. Social Science & Medicine, 314, 115460. <https://doi.org/10.1016/j.socscimed.2022.115460>. [CC BY 4.0](https://creativecommons.org/licenses/by/4.0/)

000: not in work, not in education, not a parent; 0E0: in education, not a parent; W00: in work, not a parent; WE0: in work, in education, not a parent; 00P: not in work, not in education, parent; E0P: in education, parent; W0P: in work, parent; WEP: in work, in education, parent.

**Supplementary Fig. S2** State distribution plots of work-family trajectories, 18–28 years, men (n = 365)


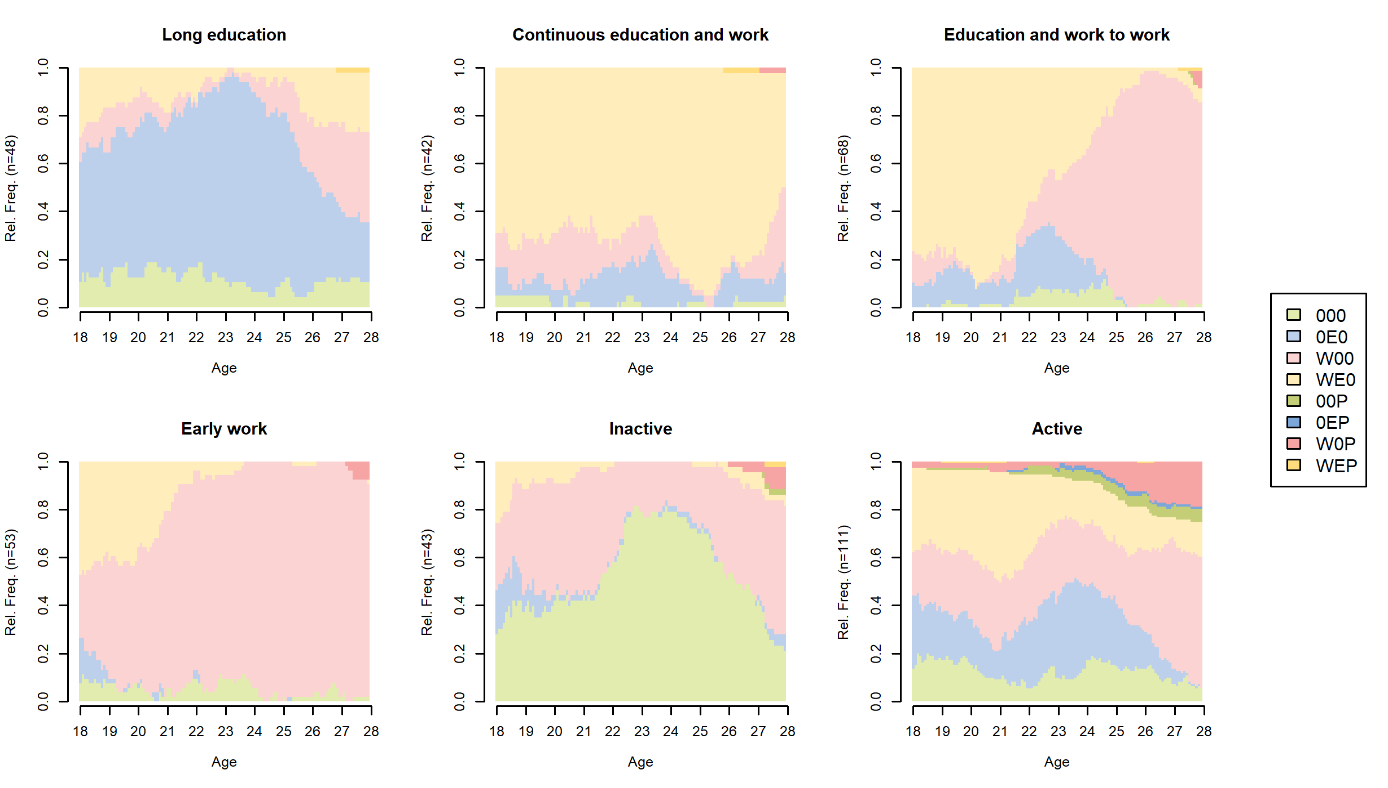


“Fig. 2. State distribution plot of work-family trajectories, 18–28 years, men (n = 365)”. From Machů, V., Veldman, K., Arends, I., & Bültmann, U. (2022). Work-family trajectories in young adulthood: Associations with mental health problems in adolescence. Social Science & Medicine, 314, 115460. <https://doi.org/10.1016/j.socscimed.2022.115460>. [CC BY 4.0](https://creativecommons.org/licenses/by/4.0/)

000: not in work, not in education, not a parent; 0E0: in education, not a parent; W00: in work, not a parent; WE0: in work, in education, not a parent; 00P: not in work, not in education, parent; E0P: in education, parent; W0P: in work, parent; WEP: in work, in education, parent.

**Supplementary Table S1** Internalising trajectories model parameter estimates, women

|  |  | Work-family life course |  |  |  |  |  | Missing [%] |
| --- | --- | --- | --- | --- | --- | --- | --- | --- |
|  | Age | Long education (n = 178) | Continuous education and work (n = 62) | Education and work to work (n = 113) | Early work (n = 96) | Inactive (n = 85) | Active with children (n = 93) |  |
| Mean | 11 | 0.385 | 0.431 | 0.397 | 0.395 | 0.413 | 0.397 | 2.1 |
|  | 13.5 | 0.382 | 0.394 | 0.400 | 0.438 | 0.400 | 0.371 | 2.1 |
|  | 16 | 0.382 | 0.367 | 0.394 | 0.426 | 0.430 | 0.370 | 8.5 |
|  | 19 | 0.306 | 0.305 | 0.282 | 0.268 | 0.358 | 0.263 | 2.6 |
|  | 22 | 0.321 | 0.317 | 0.310 | 0.288 | 0.391 | 0.265 | 1.1 |
|  | 26 | 0.395 | 0.369 | 0.368 | 0.353 | 0.466 | 0.282 | 3.2 |
|  | 29 | 0.369 | 0.314 | 0.315 | 0.326 | 0.452 | 0.278 | 0 |
| Random-intercept variance |  | 0.031 | 0.026 | 0.037 | 0.033 | 0.043 | 0.026 |  |
| Residual variance | 11 | 0.042 | 0.041 | 0.044 | 0.049 | 0.053 | 0.053 |  |
|  | 13.5 | 0.043 | 0.028 | 0.033 | 0.043 | 0.056 | 0.043 |  |
|  | 16 | 0.023 | 0.029 | 0.029 | 0.027 | 0.042 | 0.040 |  |
|  | 19 | 0.028 | 0.036 | 0.027 | 0.025 | 0.036 | 0.026 |  |
|  | 22 | 0.034 | 0.037 | 0.027 | 0.016 | 0.040 | 0.026 |  |
|  | 26 | 0.038 | 0.035 | 0.030 | 0.041 | 0.068 | 0.026 |  |
|  | 29 | 0.042 | 0.033 | 0.042 | 0.054 | 0.046 | 0.037 |  |

**Supplementary Table S2** Externalising trajectories model parameter estimates, women

|  |  | Work-family life course |  |  |  |  |  | Missing [%] |
| --- | --- | --- | --- | --- | --- | --- | --- | --- |
|  | Age | Long education (n = 178) | Continuous education and work (n = 62) | Education and work to work (n = 113) | Early work (n = 96) | Inactive (n = 85) | Active with children (n = 93) |  |
| Mean | 11 | 0.209 | 0.236 | 0.268 | 0.248 | 0.262 | 0.267 | 1.6 |
|  | 13.5 | 0.237 | 0.263 | 0.262 | 0.281 | 0.321 | 0.312 | 1.6 |
|  | 16 | 0.254 | 0.267 | 0.274 | 0.313 | 0.348 | 0.343 | 7.8 |
|  | 19 | 0.207 | 0.212 | 0.193 | 0.222 | 0.241 | 0.229 | 2.6 |
|  | 22 | 0.180 | 0.198 | 0.183 | 0.178 | 0.221 | 0.180 | 1.1 |
|  | 26 | 0.198 | 0.166 | 0.190 | 0.194 | 0.233 | 0.161 | 3.2 |
|  | 29 | 0.178 | 0.141 | 0.149 | 0.157 | 0.216 | 0.161 | 0 |
| Random-intercept variance |  | 0.014 | 0.009 | 0.014 | 0.014 | 0.016 | 0.014 |  |
| Residual variance | 11 | 0.016 | 0.016 | 0.024 | 0.032 | 0.024 | 0.027 |  |
|  | 13.5 | 0.018 | 0.019 | 0.012 | 0.027 | 0.027 | 0.029 |  |
|  | 16 | 0.018 | 0.021 | 0.017 | 0.028 | 0.025 | 0.043 |  |
|  | 19 | 0.017 | 0.017 | 0.010 | 0.015 | 0.019 | 0.021 |  |
|  | 22 | 0.010 | 0.024 | 0.009 | 0.009 | 0.021 | 0.010 |  |
|  | 26 | 0.015 | 0.010 | 0.015 | 0.009 | 0.016 | 0.010 |  |
|  | 29 | 0.017 | 0.014 | 0.009 | 0.013 | 0.019 | 0.014 |  |

**Supplementary Table S3** Internalising trajectories model parameter estimates, men

|  |  | Work-family life course |  |  |  |  |  | Missing [%] |
| --- | --- | --- | --- | --- | --- | --- | --- | --- |
|  | Age | Long education (n = 48) | Continuous education and work (n = 42) | Education and work to work (n = 68) | Early work (n = 53) | Inactive (n = 43) | Active (n = 111) |  |
| Mean | 11 | 0.385 | 0.349 | 0.353 | 0.390 | 0.336 | 0.311 | 1.4 |
|  | 13.5 | 0.360 | 0.313 | 0.255 | 0.311 | 0.247 | 0.236 | 1.6 |
|  | 16 | 0.281 | 0.256 | 0.202 | 0.267 | 0.237 | 0.198 | 10.1 |
|  | 19 | 0.262 | 0.183 | 0.158 | 0.170 | 0.211 | 0.179 | 3.0 |
|  | 22 | 0.295 | 0.241 | 0.170 | 0.198 | 0.223 | 0.204 | 3.0 |
|  | 26 | 0.293 | 0.330 | 0.225 | 0.258 | 0.280 | 0.259 | 10.1 |
|  | 29 | 0.294 | 0.287 | 0.203 | 0.251 | 0.296 | 0.236 | 0.0 |
| Random-intercept variance |  | 0.034 | 0.029 | 0.019 | 0.017 | 0.027 | 0.023 |  |
| Residual variance | 11 | 0.030 | 0.046 | 0.048 | 0.053 | 0.043 | 0.046 |  |
|  | 13.5 | 0.031 | 0.038 | 0.034 | 0.031 | 0.015 | 0.024 |  |
|  | 16 | 0.010 | 0.019 | 0.015 | 0.026 | 0.028 | 0.015 |  |
|  | 19 | 0.014 | 0.017 | 0.012 | 0.013 | 0.035 | 0.012 |  |
|  | 22 | 0.037 | 0.016 | 0.016 | 0.012 | 0.031 | 0.025 |  |
|  | 26 | 0.026 | 0.035 | 0.041 | 0.022 | 0.041 | 0.033 |  |
|  | 29 | 0.038 | 0.019 | 0.026 | 0.028 | 0.054 | 0.038 |  |

**Supplementary Table S4** Externalising trajectories model parameter estimates, men

|  |  | Work-family life course |  |  |  |  |  | Missing [%] |
| --- | --- | --- | --- | --- | --- | --- | --- | --- |
|  | Age | Long education (n = 48) | Continuous education and work (n = 42) | Education and work to work (n = 68) | Early work (n = 53) | Inactive (n = 43) | Active (n = 111) |  |
| Mean | 11 | 0.292 | 0.319 | 0.313 | 0.357 | 0.301 | 0.276 | 0.8 |
|  | 13.5 | 0.280 | 0.289 | 0.291 | 0.340 | 0.253 | 0.254 | 1.1 |
|  | 16 | 0.270 | 0.295 | 0.287 | 0.345 | 0.302 | 0.286 | 9.3 |
|  | 19 | 0.241 | 0.216 | 0.187 | 0.247 | 0.230 | 0.216 | 3.0 |
|  | 22 | 0.216 | 0.235 | 0.166 | 0.260 | 0.198 | 0.191 | 3.0 |
|  | 26 | 0.184 | 0.215 | 0.157 | 0.245 | 0.236 | 0.193 | 10.1 |
|  | 29 | 0.194 | 0.231 | 0.142 | 0.210 | 0.207 | 0.163 | 0.0 |
| Random-intercept variance |  | 0.026 | 0.017 | 0.013 | 0.029 | 0.018 | 0.015 |  |
| Residual variance | 11 | 0.042 | 0.039 | 0.032 | 0.064 | 0.028 | 0.036 |  |
|  | 13.5 | 0.035 | 0.026 | 0.021 | 0.044 | 0.010 | 0.026 |  |
|  | 16 | 0.013 | 0.021 | 0.012 | 0.028 | 0.033 | 0.024 |  |
|  | 19 | 0.016 | 0.021 | 0.011 | 0.013 | 0.036 | 0.017 |  |
|  | 22 | 0.013 | 0.014 | 0.012 | 0.020 | 0.018 | 0.012 |  |
|  | 26 | 0.006 | 0.015 | 0.007 | 0.013 | 0.036 | 0.011 |  |
|  | 29 | 0.023 | 0.017 | 0.011 | 0.016 | 0.038 | 0.011 |  |
